# Pandemic (H1N1) 2009 and HIV Co-infection

**DOI:** 10.3201/eid1610.100341

**Published:** 2010-10

**Authors:** Enrico Barchi, Francesca Prati, Maria Parmeggiani, Maria Luisa Tanzi

**Affiliations:** Author affiliations: Ospedale Santa Maria Nuova, Reggio Emilia, Italy (E. Barchi, F. Prati, M. Parmeggiani);; Università degli Studi di Parma, Parma, Italy (M.L. Tanzi)

**Keywords:** Pandemic (H1N1) 2009, HIV-1, encephalopathy, Italy, letter

**To the Editor:** We report a case of pandemic (H1N1) 2009 infection in a man with serologic evidence of HIV-1 infection. The clinical course was complicated by lung and brain involvement (respiratory failure and lethargy), severe leukopenia, and thrombocytopenia, but complications resolved after treatment with oseltamivir (150 mg 2×/d).

In November 2009, a 47-year-old man who had received a diagnosis of hepatitis C infection 8 months earlier sought treatment at Ospedale Santa Maria Nuova, Reggio Emilia, Italy. He had a 3-day history of fever, dry cough, and drowsiness. Eight days before being admitted, the man had resided in the hospital’s inpatient detoxification unit, in which at least 10 influenza-like cases had been recorded. While in the detoxification unit, he had received methadone, 50 mg 1×/d. Computed tomography images of the brain and radiographs of the chest were normal; ultrasound examination showed upper lobe consolidation of the left lung. Hematochemistry showed high creatine phosphokinase levels, leukocyte count of 1,380 cells/mm^3^ (reference range 4,000–10,000 cells/mm^3^), thrombocyte count of 34,000 cells/mm^3^ (reference range 150,000–450,000 cells/mm^3^), partial pressure of oxygen 56 mm Hg, and partial pressure of carbon dioxide 53 mm Hg. Urinalysis results were negative for heroin, cocaine, and alcohol; cerebrospinal fluid (CSF) analysis results were within normal limits. Thrombocyte count returned to reference range after 2 days, and leukocyte count improved but remained <3,500 cells/mm^3^ for 3 weeks. After admission to hospital, the man became lethargic and received noninvasive continuous positive airway pressure ventilation and treated with oseltamivir (150 mg 2×/d for 5 d), as well as with ceftriaxone, and levofloxacin. Reverse transcription–PCR on a throat swab confirmed influenza subtype H1N1 infection; blood cultures and urine were negative for pneumococcus, and *Legionella* spp. antigens. In addition, PCR of CSF for enterovirus and herpesvirus had negative results. The patient needed respiratory support for 4 days, after which his mental status and blood gases returned to reference levels. He was discharged from the hospital 2 weeks later.

On day 3 after admission, a nurse was accidentally exposed to the patient’s urine through her eye. An ELISA was positive for HIV infection. Negative results for confirmatory Western blot tests on days 5, 15, and 23 showed the p24 and p41 bands; HIV RNA was >6 million copies/mL, CD4 lymphocytes 51% (reference range 29%–59%). Reverse transcription–PCR for influenza subtype H1N1 performed 2 months later on a stored CSF sample gave a negative result; PCR for HIV of the same sample indicated 25,000 copies/mL. In mid-December, because of a further drop in CD4 lymphocytes to 17% (214 cells/mm^3^) and blood HIV RNA of 2.8 million copies/mL, the patient started highly active antiretroviral therapy and is being followed up as an outpatient.

Influenza (H1N1) and primary HIV infection share many signs and symptoms, such as fever, cough, sore throat, joint or limb pain, and diarrhea. The infections also share uncommon complications of the central nervous system (CNS); e.g., drowsiness, coma, and seizures. We cannot confirm that CNS involvement in the patient reported here was caused primarily by pandemic (H1N1) 2009, as suggested by influenza-like symptoms and the apparent effect of oseltamivir. Nor can we attribute CNS involvement to primary infection with HIV-1 (*1*); CSF results within normal limits and PCR negative for influenza subtype H1N1 do not rule out a causal relationship with pandemic (H1N1) 2009. In fact, the few cases of pandemic (H1N1) 2009 encephalopathy described show similar characteristics among children and adults (*2**–**4*). Alternatively, some authors have attributed HIV in CSF to brain inflammation and damage (*5**,**6*). The severe leukocytopenia and thrombocytopenia in our patient have not been described, even in complicated influenza subtype H1N1 infections (*7*). Because lymphopenia and mild thrombocytopenia are the usual findings, we believe that they probably resulted from HIV-1 or the effect of both viruses.

HIV seroconversion may initially occur during an acute febrile illness resembling influenza, and CNS involvement can complicate both infections. During an epidemic, acute HIV infection should also be considered (*8*). Less frequently, as in the patient described above, the 2 infections can occur simultaneously. History of recent risk behavior for blood exposure and severe leukocytopenia and thrombocytopenia should alert clinicians to other causes and prompt them to offer an HIV test to the patient.
